# Pietra Leccese and Other Natural Stones in Puglia Region: A New Category of Building Materials for Radiation Protection?

**DOI:** 10.3390/ijerph182111213

**Published:** 2021-10-26

**Authors:** Giuseppe La Verde, Adelaide Raulo, Vittoria D’Avino, Giovanni Paternoster, Vincenzo Roca, Marco La Commara, Mariagabriella Pugliese

**Affiliations:** 1 Dipartimento di Fisica “Ettore Pancini” Università degli Studi di Napoli Federico II, Via Cinthia ed. 6, 80126 Napoli, Italy; vdavino@na.infn.it (V.D.); paternoster@na.infn.it (G.P.); pugliese@na.infn.it (M.P.); 2 Istituto Nazionale di Fisica Nucleare, INFN Sezione di Napoli Via Cinthia ed. 6, 80126 Napoli, Italy; marco.lacommara@na.infn.it; 3 LB Business Services SRL, 00135 Roma, Italy; adelaide.raulo@gmail.com; 4 Dipartimento di Matematica e Fisica, Università della Campania “L. Vanvitelli”, Viale Lincoln 5, 81100 Caserta, Italy; vroca222@gmail.com; 5 Dipartimento di Farmacia, Università degli Studi di Napoli Federico II, Via Domenico Montesano, 49, 80131 Napoli, Italy

**Keywords:** building materials, external exposure, gamma spectroscopy, XRF spectroscopy, I_γ_ index, natural stone

## Abstract

In this paper, an in-depth and systematic study of the radiological characterization of three types of Puglia region natural limestones (*Pietra Leccese*, *Pietra Mazzara* and *Carparo*) was carried out. The investigation was performed by XRF spectroscopy for a chemical analysis, and gamma spectroscopy of the specific activity concentration of natural radionuclides ^226^Ra, ^232^Th, and ^40^K. Although the limestone does not fall within the category included by Italian Legislative Decree 101/2020, the gamma index was calculated using the results of the gamma spectroscopy measurements. For *Pietra Mazzara* and *Carparo* stones, the gamma index was found to be less than the reference value; conversely *Pietra Leccese* was found to be higher. To obtain a more complete evaluation of the external exposure, radium equivalent activity and external radiation hazard were calculated for all analyzed stones. The results suggest the need to broadly consider the radiological risk for these stones, and for limestone more generally, when used as a building material.

## 1. Introduction

Background radiation of natural origin provides the greatest contribution to the external dose of the world population [1,2]. The major natural sources are ^238^U, ^232^Th, with their progeny, and ^40^K present in the soil, sands and rocks, whose abundance and distribution depend on the local geology of each region of the world. Gamma radiation can occur in both outdoor and indoor spaces due to the use of natural materials in dwellings. Prolonged exposure to low doses of radiation can have a negative impact on human health [3,4]. For this reason, it is interesting to evaluate the concentration of radiation emitters in building materials (BM) of natural origin used in dwellings, since people spend most of their life inside [1,5].

In 1999, the European Commission dealt with the issue of BM radiation protection for the first time in Radiation Protection 112 (RP112) [6]. This guidance also introduced a screening tool for the identification of BM potential radiological interest: the I gamma index (I_γ_). The importance of this aspect is confirmed by the fact that RP112 itself will constitute the reference document for the Directive 59/2013 EURATOM [7] that requires the pre-characterization of construction materials (Annex XIII, Directive 59/2013 EURATOM) to limit human exposure and health effects, providing a reference level for effective dose and the radionuclides to be measured (Art. 75 and Annex VIII, Directive 59/2013 EURATOM).

In 2020, Italy implemented the Directive 59/2013 EURATOM with Legislative Decree n. 101 [8], making mandatory the indications of the Directive (Art. 29) for natural materials, i.e., alum shale or BM of igneous origin, and for materials that incorporate residues from industries that process natural radioactive materials (Annex II, Legislative Decree 101/2020). In this context, it becomes even more important to carry out wide-ranging investigations on BM.

The aim of the present study is to investigate more deeply the preliminary results obtained in [9] by measuring the natural stones’ activity concentration by high resolution gamma ray spectroscopy in the stones: *Pietra Leccese*, *Pietra Mazzara* and *Carparo*.

The physical and geological characterization of the analyzed stones has been reported in more detail in [10]. The origin of all three stones is calcareous, in fact, they consist of concretionary deposits of calcareous waters and are made up mainly of calcite or dolomite with traces of fossil plants or shells. To confirm this composition, and to have more complete information additionally from the chemical point of view, XRF characterization was performed.

Notwithstanding that this category of natural stones was not included in the indicative list of BM (Annex II, Legislative Decree 101/2020), it was considered appropriate to deepen the characterization of these stones, considering their massive diffusion in Apulian architecture. 

Due to the peculiar characteristics of these stones used in construction, indoor radon concentrations have already been measured in dwellings of the Puglia region [11] and remedial actions were implemented to reduce the concentration of radon in those homes where the value exceeded that suggested by European legislation [12]. Italy has a different, varied and peculiar territory from a geological point of view, which is why even the distribution of radon is not uniform. Some sites are of calcarenite origin, such as Puglia; others of volcanic origin, such as Campania, are of particular interest for the monitoring and management of potential radon prone areas [13,14,15]. 

Although there is no regulatory requirement, the same approach was applied as reported by [8] for standard building materials for dose evaluation due to gamma exposure, by calculating the I_γ_ index.

The measured radionuclides activity concentration value was compared with those contained in the ISTISAN report 17/36 [16]. 

Finally, radium equivalent activity (Ra_eq_) and external radiation hazard (H_ex_) were calculated. These two parameters are not mentioned in the Italian legislative decree, but they are necessary for a more complete assessment of gamma radiation exposure [1].

## 2. Materials and Methods

### 2.1. Sample Collection Site

Puglia is a singular case in Italian geological history. Starting from the Middle Pleistocene, the Apennine subduction, which until then had been uniform throughout the Italian territory, underwent an alteration: the Apulian foreland began to rise, in contrast to the northern one of the central Adriatic where subsidence continued regularly [17]. 

The Apulian limestones represent the most abundant component of the backbone of the Salento peninsula. They are mainly carbonate sediments with a bioclastic predominance, weakly cemented, characteristic of shallow temperate marine waters and shorelines.

In this region there are several quarries for the extraction of limestones [18], however, based on the geological age, materials with different chemical compositions and physical properties can be extracted [19].

Among the main limestones, there are:

1. Fine-grained, homogeneous, mostly porous, and scarcely tenacious organogenic marly limestones characterized by the presence of glauconite granules: *Pietra Leccese* is mainly used as ornamental and decorative stone; and
2. Predominantly organogenic limestones with fine to coarse grain and varying degrees of compactness, porosity, and toughness; they are sometimes associated with sandy-clayey deposits. They are defined as tuff and can be divided into two types: finer-grained tuff is very porous, light and not very resistant to compression, such as *Pietra Mazzara,* which is used in the construction of roof vaults; coarser-grained tuff is more compact, heavy, and resistant, such as *Carparo*, and is used for the load-bearing structures of the building or as cladding material.

Another use of tuff is in powdered form. They can be used to produce mortars, based on the percentage of CaCO_3_, for the manufacture of cement.

**Figure 1:**
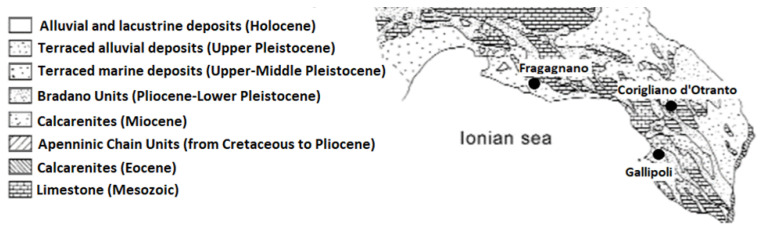
Geological map of Salento area with geographic localization of quarries.

The sampling was conducted based on the 2018 updated quarry census of the Puglia region, and on the basis of the specific extraction basins for each of the three types of stone. Therefore, ten *Pietra Leccese* stone samples were taken from Corigliano d’Otranto quarries, ten *Pietra Mazzara* from Fragagnano, and ten samples of *Carparo* stone from Gallipoli. The sites of these quarries match the areas of geological characterization reported by Ricchetti [20] as can be seen from the graphic reworking in Figure 1.

### 2.2. Sample Preparation

**Figure 2:**
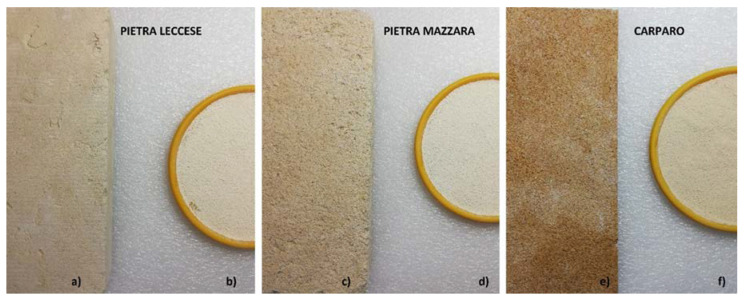
Picture of stones’ samples of *Pietra Leccese* (**a**), *Pietra Mazzara* (**c**) and *Carparo* (**e**) and respective powder (**b**–**f**).

For sample preparation, UNI EN ISO 18589-2:2015 was applied [21]. The samples (Figure 2a–e) were prepared by reducing bricks to powder by grinding (PM 100 Retsch) and sieving, drying in an oven (DIGITRONIC Selecta 2005141) at 105 °C for two hours and homogenizing the powder. The powder (Figure 2b–f) was weighted and sealed in a Marinelli beaker for 4 weeks to allow 226Ra and gamma daughters to reach secular equilibrium.

The treated samples were analyzed by gamma spectroscopy, while for XRF analysis the powder was homogenized and pressed in 10 mm diameter and 2 mm thickness tablets.

### 2.3. X-ray Fluorescence Spectroscopy Measurements

The chemical-physical analysis for the study of these materials was carried out using X-ray fluorescence with a portable device.

**Figure 3:**
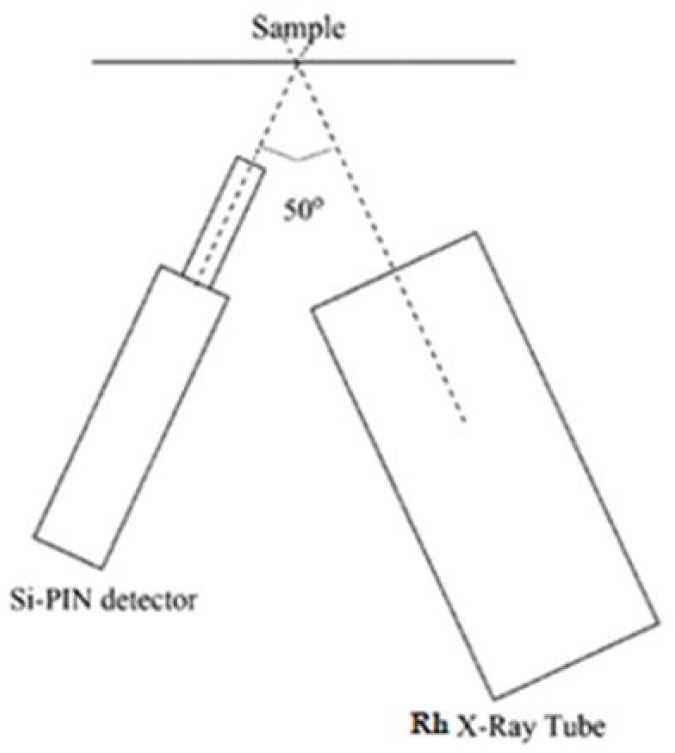
Schematic drawing of apparatus for X-ray fluorescence spectroscopy.

XRF measurements were performed using a rhodium anode X-ray generator, powered with a voltage of 40 kV, with a current of 0.2 mA; a FAST SDD^®^ X-ray detector (X-123FASTSDD by Amptek^®^ Inc., Bedford, MA 01730, USA) with a resolution of 125 eV at 5.9 keV; and a standard electronic chain for power supply and signal processing [22]. The layout of the XRF apparatus is shown as a schematic drawing in Figure 3.

The detector was equipped with an 8 µm beryllium window which, combined with helium flushing, allowed the detection of light elements starting from aluminium. The exposure time was 300 s for each analysis.

The quantitative analysis was carried out with the bAxil™ (X-ray Analysis Software, CANBERRA, Benelux, Belgium,) commercial software package. For calibration, IAEA certified standards (IAEA/Soil-7, IAEA/SL-1, IAEA/SDM-T2, IAEA-312 ^226^Ra, ^232^Th and ^238^U in soil) were used. XRF measurements were performed at INFN CH-Net infrastructure.

### 2.4. Gamma-Ray Spectroscopy Measurements

Coaxial High Purity Germanium (HPGe ORTEC^®^ AMETEK, Oak Ridge, TN, USA) detector, model GMX-45P4ST with beryllium windows, which allowed reaching good sensitivity also at energy lower than 100 keV, was used. The properties were 48% relative efficiency and 2.16 keV at 1.33 MeV measured energy resolution. 

The spectra were acquired by ORTEC^®^ DSPEC-LF unit plus MCA Emulator software, and analyzed with GammaVision Spectrum Analysis Software. A 10 cm thick lead shield prevented background count due to external environmental radiation. The minimum detectable activity (MDA) of the system has been estimated with a 95% confidence level [23]. 

Acquisition time was 86,400 s (i.e., 24 h) for both background and sample measurements, in order to obtain a good statistical counting for each sample.

The gamma-ray spectra were analyzed taking into account the ^238^U and ^232^Th decay chains, and ^40^K. The full energy peaks used for the activity concentration determinations were: 63.2 keV and 92.5 keV for ^234^Th (^238^U), 186 keV for ^226^Ra, 46.50 keV for ^210^Pb (^238^U), 911.1 keV and 968.9 keV for ^228^Ac (^232^Th). The gamma-ray at 1461 keV is associated with the ^40^K decay. The interference between gamma lines of 186.0 keV of ^226^Ra and 185.7 keV of ^235^U was solved by sharing the areas of the respective peaks in function of each relative branching ratio with the previously described peak fit program.

The combined standard uncertainty was calculated by considering the error associated with counting, gamma emission probability, energy and efficiency calibration, and sample mass as reported in [24].

### 2.5. Gamma-Index

Some indexes dealing with the assessment of the excess gamma radiation from building materials (frequently called ‘‘gamma-indexes’’ or ‘‘external-indexes’’) have been proposed [25,26]. 

In this study, the I_γ_ was calculated as adopted by the European Commission RP112, by Euratom 59/2013 Annex VIII and finally implemented by Italian Decree, Annex II.

The I_γ_ is described by Equation (1):

(1) $$I_{\gamma }=\frac{C_{\mathrm{Ra}}}{300{\;\mathrm{Bq}\;\mathrm{kg}}^{-1}}+\frac{C_{\mathrm{Th}}}{200{\;\mathrm{Bq}\;\mathrm{kg}}^{-1}}+\frac{C_{K}}{3000{\;\mathrm{Bq}\;\mathrm{kg}}^{-1}}$$

where C_Ra_, C_Th_, C_K_ are the ^226^Ra, ^232^Th and ^40^K activity concentrations (Bq kg^−1^), respectively, in the building material. For more information about parameter values used in deriving this gamma-index, see the work of Markkanen [27].

### 2.6. Radium Equivalent Activity and External Radiation Hazard

The gamma radiation exposure was also defined by calculation of the radium equivalent activity (Ra_eq_) index (see Equation (2)), which was based on the assumption that 370 Bq kg^−1^ of ^226^Ra, 259 Bq kg^−1^ of ^232^Th, and 4810 Bq kg^−1^ of ^40^K, produce the same gamma-ray dose rate.

(2) $${\mathrm{Ra}}_{\mathrm{eq}}=A_{Ra}+1.43A_{Th}+0.077A_{K}$$

Ra_eq_ is related to both the external γ dose and the internal α dose due to inhalation of radon and its progeny. This work focused on external gamma exposure, and then the external radiation hazard (*H_ex_*) was calculated according to two different models:

1. A model for a room with infinitely thick walls without windows and doors, as reported in the following Equation (3) [1]:

(3) $$\;H_{ex}=\frac{A_{Ra}}{370}+\frac{A_{Th}}{259}+\frac{A_{K}}{4810}$$

2. A model for a room with doors and windows, described by Equation (4), for which the presence of doors and windows and a consequent ventilation would have the exposure to radiation [28]:

(4) $$H_{ex}=\frac{A_{Ra}}{740}+\frac{A_{Th}}{518}+\frac{A_{K}}{9620}$$

## 3. Results and Discussion

### 3.1. XRF—Measurement Results

The samples’ chemical composition obtained by XRF results are reported in Table 1:

The abundance of CaO in all three samples confirms the calcareous nature of these stones, as also reported in the geological characterization document [19].

### 3.2. Activity Concentration Determined by Gamma Measurements

The BM activity concentration results of ^226^Ra, ^232^Th, and ^40^K are shown in Table 2:

As can be seen in Table 2, the measured minimum ^226^Ra activity concentration was 30 ± 2 Bq kg^−1^ in the *Carparo* samples, and the maximum was 406 ± 21 Bq kg^−1^ in the *Pietra Leccese* samples. 

The ^232^Th activity concentration minimum observed was 1.0 ± 0.8 Bq kg^−1^ in the *Pietra Mazzara* samples, and the maximum was 7.1 ± 3.2 Bq kg^−1^ in the *Carparo* samples. 

The ^40^K activity concentration ranged from 6.8 ± 1.2 Bq kg^−1^ in *Pietra Mazzara* up to 30.5 ± 3.1 Bq kg^−1^ in *Pietra Leccese*. 

The natural radioactivity level measured in the samples, was compared with the values reported for limestone in the ISTISAN report (see Table 3) [16]. Even if the analyzed samples belonged to the same category, the results of the gamma spectrometry were partially different, likely due to the peculiar geological origin of the stones [17], compared with those of the references [29,30].

As can be seen in Table 2, the activity concentration of ^226^Ra in *Pietra Leccese* and *Pietra Mazzara* stones was higher than the values reported in Table 3, while comparable value was found for *Carparo*. The other radionuclides were close to each other.

On the other hand, comparing present results with the preliminary study [9], the ^226^Ra in *Pietra Mazzara* was higher; and the ^232^Th and ^40^K average activity concentration for all samples were similar. 

### 3.3. I_γ_ Index, Raeq and Hex

The results obtained for the analyzed materials are reported in Table 4:

As stated by [8] (Art. 29 and Annex II), the I_γ_ index value equals 1 can be used as a conservative screening tool for identifying materials that may imply the exceedance of the effective dose limit of 1 mSv/year. If I_γ_ > 1, a material can still be used, but it must be proved that the dose limit is not exceeded in conditions under which the material is intended to be used (e.g., for local use as *Pietra Leccese* in the Apulian area). It is also possible to apply a dose criterion that takes into account the typical ways and quantities in which the material is used in a building. For materials used in bulk amounts, e.g., bricks, the dose criterion is 0.3 mSv/year, and 1 mSv/year, for I_γ_ ≤ 0.5 and I_γ_ ≤ 1, respectively. On the contrary, for superficial and other materials with restricted use such as tiles and boards, the dose criterion is 0.3 mSv/year, and 1 mSv/year, for I_γ_ ≤ 2 and I_γ_ ≤ 6, respectively. Therefore, *Pietra Leccese* is more widely used as an external cladding of walls, or decoration of the building; *Pietra Mazzara* and *Carparo* are most often used as bricks to construct the building.

Ra_eq_ should not exceed the value of 370 Bq kg^−1^, equal to an effective dose of 1 mSv/year for the population [1], which is also the reference level applicable to external exposure to gamma radiation emitted by BM indoors, in addition to external exposure outdoors (art.29) [8]. *Pietra Leccese* and *Pietra Mazzara* have values higher than the limit of 370 Bg kg^−1^, 1153 and 749 Bq kg^−1^ respectively and this is consistent with the ^226^Ra content reported in Table 2. Therefore, it is necessary to calculate the H_ex_.

The H_ex_ value should be less than unity to avoid an effective dose greater than 1 mSv/year. In this case *Pietra Leccese* and *Pietra Mazzara* have H_ex_ values less than or equal to 1 according to both models (Equations (3) and (4)).

## 4. Conclusions

Radiation protection concerning buildings materials is going to be of great interest as indicated in [6,7,8].

In this study, thirty samples of different natural materials were analyzed: *Pietra Leccese*, *Pietra Mazzara* and *Carparo*. The different kinds of materials were sampled in specific quarries in Puglia Region to determine a complete characterization for each. 

The XRF analysis and gamma spectroscopy measurements were performed to obtain representative chemical–mineralogical characterization and natural radioactivity concentration, respectively. 

The I_γ_ index of *Pietra Leccese*, was slightly higher than the reference value. Ra_eq_ of *Pietra Leccese* and *Pietra Mazzara* was higher than 370 Bq kg^−1^; however, for these two types of BM, H_ex_ was ≤1 which is the limit value which ensures an exposure of less than 1 mSv/year.

All the results obtained represent an in-depth study of a preliminary study, and confirm the importance of considering the issue of radioprotection for the safe use of *Pietra Leccese* and other limestones that have the same application.

The results of this study offer experimental data that could update the ISTISAN database, which does not contain limestones from the Puglia Region.

## Figures and Tables

**Table 1:** Stones’ chemical composition: XRF analysis.

		MDL	*Pietra Leccese*	*Pietra Mazzara*	*Carparo*
Al_2_O_3_	%	5	n.d.	n.d.	n.d.
SiO_2_	%	2	5.2 ± 0.9	2.0 ± 0.8	1.9 ± 0.9
K_2_O	%	0.2	0.35 ± 0.03	0.22 ± 0.03	0.17 ± 0.03
CaO	%	0.1	99.7 ± 0.3	96.2 ± 0.2	96.5 ± 0.3
TiO_2_	ppm	300	702 ± 171	312 ± 148	269 ± 103
MnO	ppm	150	160 ± 45	602 ± 57	435 ± 63
Fe_2_O_3_	%	0.01	1.31 ± 0.02	1.27 ± 0.02	1.16 ± 0.02
Co_3_O_2_	ppm	10	12 ± 3	14 ± 3	8 ± 3
NiO	ppm	40	139 ± 17	88 ± 15	64 ± 13
ZnO	ppm	60	272 ± 32	144 ± 28	141 ± 31
As_2_O_3_	ppm	50	87 ± 22	<	77 ± 30
Rb_2_O	ppm	70	<	<	<
SrO	ppm	80	2060 ± 41	1440 ± 35	1500 ± 41
Y_2_O_3_	ppm	150	289 ± 58	<	142 ± 61
ZrO_2_	ppm	90	157 ± 45	167 ± 42	<
Nb	ppm	150	<	<	35 ± 16
BaO	ppm	450	<	<	397 ± 144
Th	ppm	120	126 ± 52	<	155 ± 57
U	ppm	90	92 ± 33	127 ± 32	<
Legend:					
MDL	Minimum Detection Limit (IAEA: Soil7, SL1, SDMT-2, 312)				
n.d.	Not detected				
<	Less than 2 standard deviations				

**Table 2:** Average, minimum and maximum values of activity concentration of natural radionuclides in the analyzed samples.

Sample	Activity Concentration (Bq kg^−1^)								
	^226^Ra			^232^Th			^40^K		
	Average	Min	Max	Average	Min	Max	Average	Min	Max
*Pietra Leccese*	349 ± 19	313 ± 18	406 ± 21	3.2 ± 1.1	2.7 ± 1.0	3.8 ± 1.4	24.9 ± 2.7	21.1 ± 2.4	30.5 ± 3.1
*Pietra Mazzara*	213 ± 12	187 ± 11	258 ± 14	2.5 ± 1.0	1.9 ± 0.8	2.9 ± 1.3	7.5 ± 1.4	6.8 ± 1.2	7.7 ± 1.4
*Carparo*	33 ± 2	30 ± 2	38 ± 4	6.4 ± 3.0	5.6 ± 2.8	7.1 ± 3.2	12.3 ± 1.5	11.6 ± 1.4	13.0 ± 2.1

**Table 3:** Radioactivity content in limestones, ISTISAN report [12].

Building Material	Number of Samples	^226^Ra (Bq kg^−1^)			^232^Th (Bq kg^−1^)			^40^K (Bq kg^−1^)			Ref
		Mean	Max	Min	Mean	Max	Min	Mean	Max	Min	
Limestone_1	27	11	30	0.4	2			22			[29]
Limestone_2	1	65			6			46			[30]
Limestone_3	1	76			8			47			[30]

**Table 4:** Indexes results for measured samples.

Samples	I_γ_ Index	Ra_eq_ Bq kg^−1^	H_ex_ Model (3)	H_ex_ Model (4)
*Pietra Leccese*	1.19 ± 0.07	1153	1	0.5
*Pietra Mazzara*	0.75 ± 0.05	749	0.6	0.3
*Carparo*	0.14 ± 0.02	129	0.12	0.6
**Reference level**	**1**	**370**	**1**	**1**

## Data Availability

Data is contained within the article.
